# Dynamic postural balance indices can help discriminate between patients with multiple system atrophy and Parkinson's disease

**DOI:** 10.3389/fneur.2022.1089439

**Published:** 2023-01-09

**Authors:** Wei Bao, Puyu Li, Ying Yang, Kai Chen, Jun Liu

**Affiliations:** ^1^School of Mechanical Engineering, Hangzhou Dianzi University, Hangzhou, Zhejiang, China; ^2^Department of Neurology and Institute of Neurology, Ruijin Hospital, Shanghai Jiao Tong University School of Medicine, Shanghai, China

**Keywords:** Parkinson's disease (PD), multiple system atrophy (MSA), balance control, center of pressure (COP), posturography

## Abstract

**Background:**

Patients with Parkinson's disease (PD) and those with multiple system atrophy (MSA) show similar symptoms but have different clinical treatments. It will be helpful to discriminate between these two kinds of patients at an early or middle stage. The purpose of this study is to highlight the differences in posturographic characterization between patients with PD and those with MSA during quiet standing and perturbed standing.

**Methods:**

A total of clinically diagnosed 42 patients with PD and 32 patients with MSA participated in the experiment. Patients were asked to first stand on a static balance force platform and then on a dynamic balance (medial-lateral rocker) force platform to measure the center of pressure (COP) trajectory during an eyes-open (EO) state. The posturographic parameters were obtained under the two standing conditions for statistical analysis.

**Results:**

Four posturographic variables were calculated and analyzed, namely, the standard deviation of COP position (SD), sway path of COP position (SP), an elliptical area covering the 95% COP position trajectory (EA), sway path of COP position (SP), and integral area of the power spectral density at 0–0.5 Hz frequency band (PSD). Except for variable EA, the other three variables are all in the medial-lateral (ML) direction. In the static balance experiment, there were no significant differences between the four variables between patients with PD and those with MSA. However, in the dynamic balance experiment, the obtained four variables all presented significant differences between patients with PD and those with MSA.

**Conclusion:**

The dynamic posturographic variables with significant differences between patients with PD and those with MSA imply that patients with MSA have worse postural control ability in the medial-lateral (ML) direction compared to patients with PD. The obtained dynamic indices may help supplemental clinical evaluation to discriminate between patients with MSA and those with PD.

## Introduction

Patients with Parkinson's disease (PD) and those with multiple system atrophy (MSA) have many overlapping symptoms clinically, such as tremors, rigidity, bradykinesia, and posture instability, and they all have relatively large spontaneous sways when standing (1, 2). Movement disorders can be exceedingly difficult between differential diagnoses of neurodegenerative diseases, such as patients with PD and patients with MSA, who are very easily misdiagnosed (3). Accurate diagnosis is very important for correct treatment. Patients with PD are normally diagnosed by senior movement disorder specialists based on the Movement Disorder Society (MDS) diagnostic criteria for PD, which was drafted by Postuma et al. (4). MSA was diagnosed based on a novel set of diagnostic criteria from MDS, which was drafted by Wenning et al. (5). The new MSA diagnostic criteria aim at improving diagnostic accuracy, particularly in early disease stages.

Postural instability (PI) is one of the cardinal signs in the clinical diagnostic criteria of Parkinson's disease. Clinical differentiation of MSA typically relied on postural instability (PI) within 3 years of motor onset by neurologists (6). However, the differential diagnosis of neurodegenerative movement disorders can be exceedingly difficult (1). For the diagnosis of MSA, pathologically confirmed dementia with Lewy bodies (DLB) is the most common misdiagnosis, followed by progressive supranuclear palsy (PSP) and PD (7). According to Koga's report, only 62% of MSA patients' clinical diagnosis was confirmed at autopsy (7). Miki et al. researched and presented a clinicopathological study involving 203 people, of whom 78.8% were correctly diagnosed with MSA by pathological confirmation (8). In another study of surveys that confirmed MSA by autopsy, the correct diagnosis was 81.2% (9). On the contrary, the diagnosis of Parkinson's disease continues to be challenging, with misdiagnosis rates as high as 20–30% in the early stages (10). Such diagnostic inaccuracy is largely due to the failure to recognize atypical parkinsonian disorders (APDs) (10). The presence and severity of PI among patients with Parkinson's are commonly evaluated by the number of clinical tests. The most widely used tests for PI are the TUG test, the Tandem Gait test, and the pull test (11, 12). The pull test has been incorporated into the MDS-UPDRS scales (13). Tandem gait and TUG tests were used to distinguish APDs from PD (14). Though PI could not be detected in early PD patients without symptoms through these clinical tests, subclinical posture instability could be evaluated by objective assessments (15).

An objective method for the evaluation of posture stability in the clinic is to observe the patient's standing posture through posturography (16). Some subclinical PI symptoms have been shown through objective assessment of posturography in patients without any visible symptoms of PI (17). Panyakaew et al. compared the static standing PI of patients between the PD group and MSA group under eye open (EO) and eye closed (EC) conditions by analyzing the posturographic parameters (13). In the state of EC, the elliptical area covering the trajectory of the COP position in patients with MSA was larger than that in patients with PD. However, in the state of EO, there was no salient distinction. When comparing patients with PD, visual conditions have more impact on the standing posture of patients with MSA (18). But the studies comparing spontaneous sway between patients with PD and those with MSA under visual deprivation conditions have less practical meaning. In clinical practice, the standing posture of patients is normally evaluated with one eye open (EO). When patients with PD are in a state of EO, dynamic balance experiments can effectively distinguish the postural differences between patients with PD and healthy controls, which are often difficult to distinguish under the static balance condition (19). Dynamic balance experiments can also help to evaluate the motor adaptability of patients with PD (20). When a patient is standing on a dynamic force platform, the body is forced to follow the swing plane to perform a swing movement. In this disturbing environment, the standing person needs to increase their postural control to maintain body balance (21). The severity of postural sway in MSA should be shown to be worse than that of PD due to a more widespread degeneration in MSA (22). It is thus hypothesized that patients with PD and those with MSA may exhibit distinct PI features in the state of EO under dynamic standing.

The direction of PI among patients with PD and those with MSA has also been studied. Kamieniarz et al. found that the PI of patients with PD is mainly reflected in the anterior-posterior (AP) direction (2). In clinical trials, patients with MSA showed PI in the medial-lateral (ML) direction, while patients with PD did not present such features (23). Specifically, patients with MSA often have a broad stance width (24), which indicates that patients with MSA have more instability in the ML direction. Thus, it is better to use the tandem gait test for the detection of MSA (25). Patients with PD preserved their balance in the medial-lateral direction, so that many patients with PD are still able to ride their bicycles, even in the face of severe walking difficulties (26). Researchers found that patients with MSA showed a lack of coordination ability and postural defects in the ML direction in a cycling experiment (27). The previous studies demonstrated that the analysis of the posturographic characterization of patients with MSA should be focused on the ML direction, and the dynamic swing should also be in the ML direction in order to enhance the interference in the dynamic balance experiment.

The purpose of this study was to compare the differences in posturographic characterization between patients with PD patients and those with MSA under static and dynamic balance conditions at the state of EO. The obtained distinct posturographic features may help screen out patients with MSA from patients with PD during the stage of onset.

## Methods

### Participants

A total of 74 patients participated in the experiment. They were recruited from the outpatient clinics of the neurological department at Runjin Hospital in Shanghai between December 2019 and November 2020. Of them, 42 were patients with PD, and 32 were probable patients with MSA. The average age of patients with PD was 68.2 ± 7.1 years; the average age of patients with MSA was 64.8 ± 10.1 years. All patients performed assessments on the Hoehn &Yahr (H&Y) scale and the MDS-Unified Parkinson's Disease Rating Scale (MDS-UPDRS). Other examinations, such as the Berg Balance Scale (BBS) assessment, the Minimum Mental State Examination (MMSE), and the Gait and Falls Questionnaire (GFQ), were also recorded. Patients were excluded once they met one of the following conditions: H&Y stages 4–5, a history of severe neurological and psychiatric disorders, patients with significant cognitive impairment (MMSE < 24) or unable to complete the questionnaire independently, severe medical conditions preventing the patient from completing the experiment, there existing implantable materials such as intracranial stents, pacemakers, coronary stents, and cochlear implants; pregnant or lactating women. All subjects were asked not to take sedatives. All subjects were assessed at least 8 h after the last dose of anti-parkinsonian medications used to reduce the impact of dopaminergic medications (28). PD was diagnosed by senior movement disorder specialists based on the Movement Disorder Society (MDS) diagnostic criteria for PD (4). In the course of PD assessment, secondary causes (drug-induced, inflammatory, toxin-induced, and vascular parkinsonism), parkinsonism with other neurodegenerative diseases (progressive supranuclear palsy, multiple system atrophy, cortical basal ganglia degeneration, Wilson's disease, etc.), and other neurological diseases, such as stroke, were excluded. MSA was diagnosed based on the diagnostic criteria for MSA, which were drafted by Gilman et al. in 2008 (6). Probable patients with MSA who participated in the experiment were categorized as MSA-P with predominant parkinsonism but no cerebellar features or as MSA-C with predominant cerebellar signs but mild or no parkinsonism (29). The baseline clinical characteristics of all subjects were recorded by two doctors with more than 10 years of clinical experience. This study was conducted in accordance with the guidelines of the Helsinki Declaration of the World Medical Association (2000) and was approved and supervised by the Ethics Committee of Shanghai Ruijin Hospital (approval No. LWEC2019017). After receiving a detailed description of the experiment, all participants signed informed consent forms. The patients' demographic information is listed in Table 1.

**Table 1:** Demographic data of the PD and MSA groups.

**Variable**	**PD (*N =* 42) (mean ±std)**	**MSA (*N =* 32) (mean ±std)**	**MSA-C (*N =* 16) (mean ±std)**	**MSA-P (*N =* 16) (mean ±std)**
Age	68.2 ± 7.1	64.8 ± 10.1	65.5 ± 10.69	64.1 ± 8.1
Disease duration (Y)	4 ± 3.28	3.3 ± 2.76	3.4 ± 2.86	3.2 ± 2.5
Sex (% Femail)	22(52%)	14(44%)	7(44%)	7(44%)
Body weight (kg)	63.2 ± 13.6	67.3 ± 12.8	65 ± 11.2	69.6 ± 13.8
Height (cm)	164 ± 7.29	166 ± 9.4	165 ± 10.4	167 ± 8.4
Body mass index	23.5 ± 2.9	24.26 ± 2.58	23.9 ± 1.58	24.62 ± 3.58
H&Y score	2.02 ± 0.57	2.6 ± 0.57	2.7 ± 0.77	2.5 ± 0.37
MDS-UPDRS score (total)	55.6 ± 21.9	76.65 ± 24.2	78.2 ± 22.2	75.1 ± 26.2
Berg Balance Scale	51.95 ± 4.1	41.23 ± 10.7	40.1 ± 11.7	42.36 ± 11.7
MMSE	27 ± 1.95	25.88 ± 2.14	25.2 ± 1.1	26.56 ± 2.1
GFQ	20 ± 11.8	22 ± 8.19	22.5 ± 6.2	21.45 ± 9.1

BBS, Berg Balance Scale; H&Y, Hoehn-Yahr Scale; MDS-UPDRS, MDS-Unified Parkinson's Disease Rating Scale; PD, Parkinson's disease; MSA, multiple system atrophy; GFQ, Gait and Falls Questionnaire.

### Device

**Figure 1:**
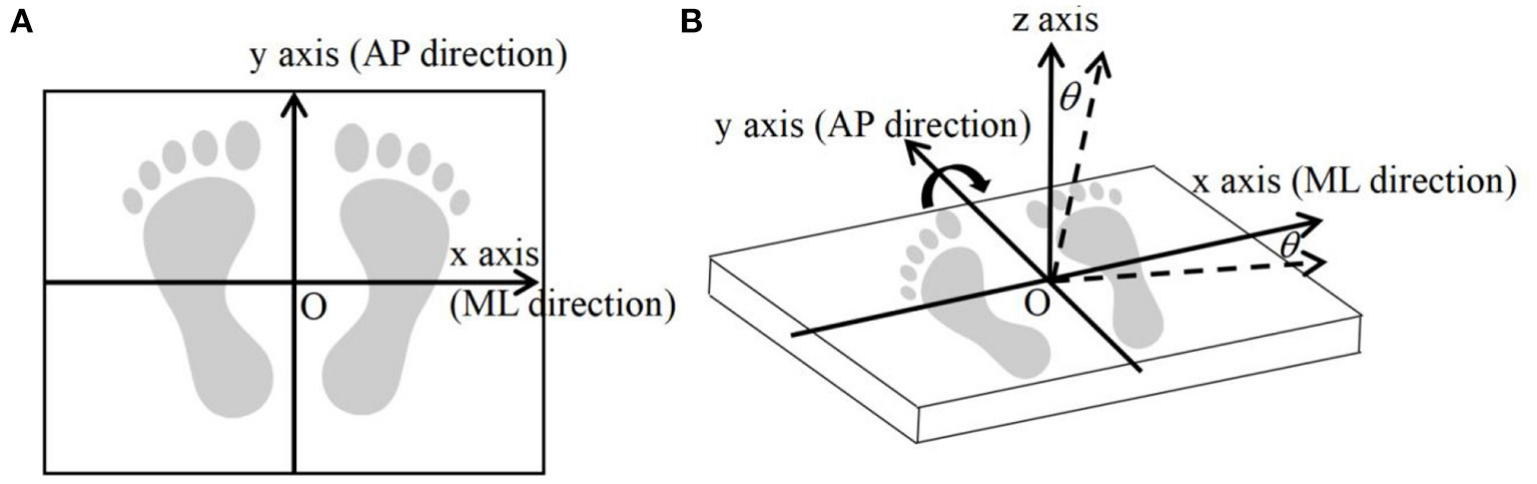
Schematic diagram of the dynamic force platform. **(A)** The patient stands still and upright on the stationary force platform with bare feet, with the *y-*axis in the AP direction and the *x-*axis in the ML direction. **(B)** The patient stands upright and barefoot on the dynamic force platform. The platform swings periodically around the *y-*axis. The patient needs to maintain body balance during the swinging process. The *z-*axis is the vertical direction when the patient stands. θ is the instantaneous swing angle of the platform. ML, medial-lateral; AP, anterior-posterior.

The patients participating in the experiment needed to stand on a platform, 60 cm × 40 cm in size. The platform is a self-developed dynamic COP measuring system comprised of an AMTI (model bp400600, Advanced Mechanical Technology Inc., MA, USA) force board, a data collector, a rocker controller, and a host computer. A detailed description of the system is provided by Chen et al. and Chang et al. (30, 31). The frequency of data acquisition is set at 500 Hz. The system can work in either a stationary or dynamic state. One state is that the platform is stationary in the horizontal plane, in which the *x-*axis is in the ML direction and the *y-*axis is in the AP direction. Another state is that the platform rotates around the *y-*axis at a small angle (within ±4°) and swings periodically along the ML direction with a frequency of 1 Hz. A schematic diagram of the dynamic force platform is shown in Figure 1.

### Experimental procedures

All of the patients participated in the static balance experiment and the dynamic balance experiment. In the static balance experiment, the patient stood barefoot naturally and with shoulder width apart, hands drooping naturally. The range of the distance between heels was 20 ± 3 cm, and the range of the angles of the feet with respect to the AP axis was 20 ± 2°. The patient gazed at a fixed eye-level mark 3 m in front. In the dynamic balance experiment, the patient's standing posture was the same as that of the static balance experiment. After the patient stood on the platform for 20 s, the platform started to swing in the ML direction. In both experiments, before recording, the patient was asked to stand for 30 s to confirm that the COP signals were maintained at a relatively stable level. The recording period was set to 70 s for each state, with the first 5 s allocated for the fade-in, the next 60 s for the formal test, and the last 5 s for the fade-out. To maintain the reliability of the collected data, each patient's test was repeated three times, and the average value was taken during the calculation of posturographic parameters. The interval between each patient's tests was 5 min, during which time the patient left the platform for relaxation. In the experiment, if the patient had difficulty maintaining balance, the experiment was terminated.

### Analysis of the COP parameters

Before the statistical analysis, the COP signal obtained by the force platform was processed by fourth-order Butterworth low-pass filtering, and the cutoff frequency was set to 10 Hz. The filtered signal was calculated by the self-developed MATLAB algorithms. In the balance test, the coordinate origin of the COP signal was the central position of the force plate. Since the starting point of each collected COP signal was different for each test, the average coordinate values of COP displacement (in the *x* and *y* directions) were taken as the offset values and were removed by the program algorithm before the actual calculation. In the dynamic balance test, the force platform swings periodically around the *y-*axis with an instantaneous swing angle θ (Figure 1). The *x* and *y* coordinates of the COP position under dynamic balance can be calculated in real-time through a coordinate transformation matrix, obtaining the instantaneous swing angle θ by counting the control pulses (31).

After obtaining the position coordinates of the COP under static balance and dynamic balance, the relevant posturographic parameters were calculated. There are many parameters related to posturographic characterization (32). In this study, four spatiotemporal variables were chosen: (1) the standard deviation (SD) of COP displacement, (2) the elliptical area covering the 95% confidence of COP position trajectory (EA) (33), (3) the sway path of COP position, and a frequency domain variable, (4) power spectral density (PSD) at 0–0.5 Hz frequency band (34). Table 2 lists the specific expressions of the four parameters. The calculation formulas for those variables were provided in the Supplementary material. Although the posturographic parameters were calculated in both the AP and ML directions, the results showed that only the parameters in the ML direction presented significant differences between the MSA and PD groups. Therefore, except for *EA*, the other three parameters listed in Table 2 are in the ML direction by default.

**Table 2:** Variables used in the analysis of the COP displacement.

**Variable**	**Description**
*SD*	Standard deviation of COP position in ML direction
*EA*	Ellipse Area covering the 95% confidence of COP position
*SP*	Sway Path of COP position in ML direction
*PSD*	Integral area of power spectral density at 0–0.5Hz frequency band in ML direction

COP, the center of pressure; ML, medial-lateral.

### Statistical analysis

After obtaining the COP signal from the tester, the statistical analysis was carried out using the IBM SPSS Statistics 25.0 software. The Shapiro–Wilk statistic was used to test the normality of the distribution of all variables. Because the data do not strictly follow a normal distribution, differences among the MSA group and PD groups were evaluated using the Mann–Whitney test for *post hoc* pair-wise tests for variables. To compare between the MSA-C, MSA-P, and PD groups, Kruskal–Wallis rank sum test was performed with Mann–Whitney tests for *post hoc* pair-wise comparisons. The significance level was set to 0.05. The correlations between the variables and the patient's clinical scale (H&Y) were calculated with Spearman's rank test. To determine the sample size, a power analysis was performed based on the previously published studies between MSA and PD. A sample size of at least 15 subjects per group was identified to detect an effect size of 0.5 with a power of 0.8 (35). A sample size of at least 15 subjects per group was needed.

## Results

We present the results of the statistical analysis of the four posturographic parameters. Subscripts _*st* and _*dy* are used to represent the conditions of static balance and dynamic balance, respectively. Table 3 lists the statistical results of the parameters obtained from the MSA patient group and the PD patient group in both the static and dynamic balance experiments. There were no significant differences between the PD and MSA groups with the four posturographic parameters in the static balance experiment. However, in the dynamic balance experiment, there were significant differences in these same parameters between the MSA group and the PD group. The variable *PSD_dy* (*p*-value = 0.006, effect size *d* value = 0.52) displays the largest difference.

**Table 3:** Statistical results of posturographic variables between PD and MSA groups.

**State of EO**	**PD (*N =* 42)**	**MSA (*N =* 32)**	**MSA vs. PD**
**Variable**	**Mean** ±**std**	**Mean** ±**std**	**P-value**
**Static**			
*SD*_*st*	0.007 ± 0.0082	0.0069 ± 0.0048	0.398
*EA_st* (cm^2^)	5.37 ± 12.25	9.1 ± 25.43	0.071
*SP*_*st* (cm)	131.48 ± 139	151 ± 95.7	0.703
*PSD*_*st*	0.599 ± 1.24	0.217 ± 0.142	0.263
**Dynamic**			
*SD*_*dy*	0.026 ± 0.0089	0.037 ± 0.014	0.001^**^
*EA_dy* (cm^2^)	52.1 ± 27.1	83.3 ± 57.13	0.002^**^
*SP*_*dy* (cm)	656.28 ± 215	835.13 ± 315.7	0.001^**^
*PSD_dy*	6.93 ± 5.47	12.96 ± 1.67	0.001^**^

^*^Indicates p-value < 0.05, ^**^Indicates p value < 0.01. PD, Parkinson's disease; MSA, multiple system atrophy; st, static; dy, dynamic.

Table 4 lists the statistical results between the MSA-C, MSA-P, and PD groups in both the static and dynamic experiments. Again, there were no significant differences between the PD, MSA-C, and MSA-P groups with all four static parameters. However, these four same parameters in dynamic balance all showed significant differences between the PD group and the MSA-C group, and two variables (*SD*_*dy* and *EA_dy*) present significant differences between the PD and the MSA-P groups.

**Table 4:** Statistical results of posturographic variables MSA-C, MSA-P, and PD groups.

**State of EO**	**PD (*N =* 42)**	**MSA-C (*N =* 16)**	**MSA-P (*N =* 16)**	**PD vs MSA-C**	**PD vs MSA-P**	**MSA-C vs MSA-P**
**Variable**	**Mean** ±**std**	**Mean** ±**std**	**Mean** ±**std**	**P-value**	**P-value**	**P-value**
**Static**						
*SD*_*st*	0.007 ± 0.0082	0.007 ± 0.002	0.0067 ± 0.008	0.554	0.128	0.254
*EA_st* (cm^2^)	5.37 ± 12.2	12.9 ± 4.78	5.29 ± 70	0.144	0.156	0.696
*SP*_*st* (cm)	131.48 ± 139	162.68 ± 44	139.9 ± 155	0.59	0.486	0.752
*PSD*_*st*	0.599 ± 1.24	0.17 ± 0.18	0.26 ± 0.67	0.135	0.16	0.8
**Dynamic**						
*SD*_*dy*	0.026 ± 0.0089	0.039 ± 0.013	0.035 ± 0.016	0.001^**^	0.035^*^	0.235
*EA_dy* (cm^2^)	52.1 ± 27.1	89.8 ± 72.8	76.8 ± 36.7	0.008^**^	0.028^*^	0.669
*SP*_*dy* (cm)	656.28 ± 215	944 ± 315	726 ± 284	0.001^**^	0.159	0.094
*PSD_dy*	6.93 ± 5.47	16.06 ± 10	9.87 ± 8	0.001^**^	0.044^*^	0.08

^*^Indicates p-value < 0.025, ^**^Indicates p-value < 0.01. PD, Parkinson's disease; MSA, multiple system atrophy; N, number; st, static; dy, dynamic.

**Figure 2:**
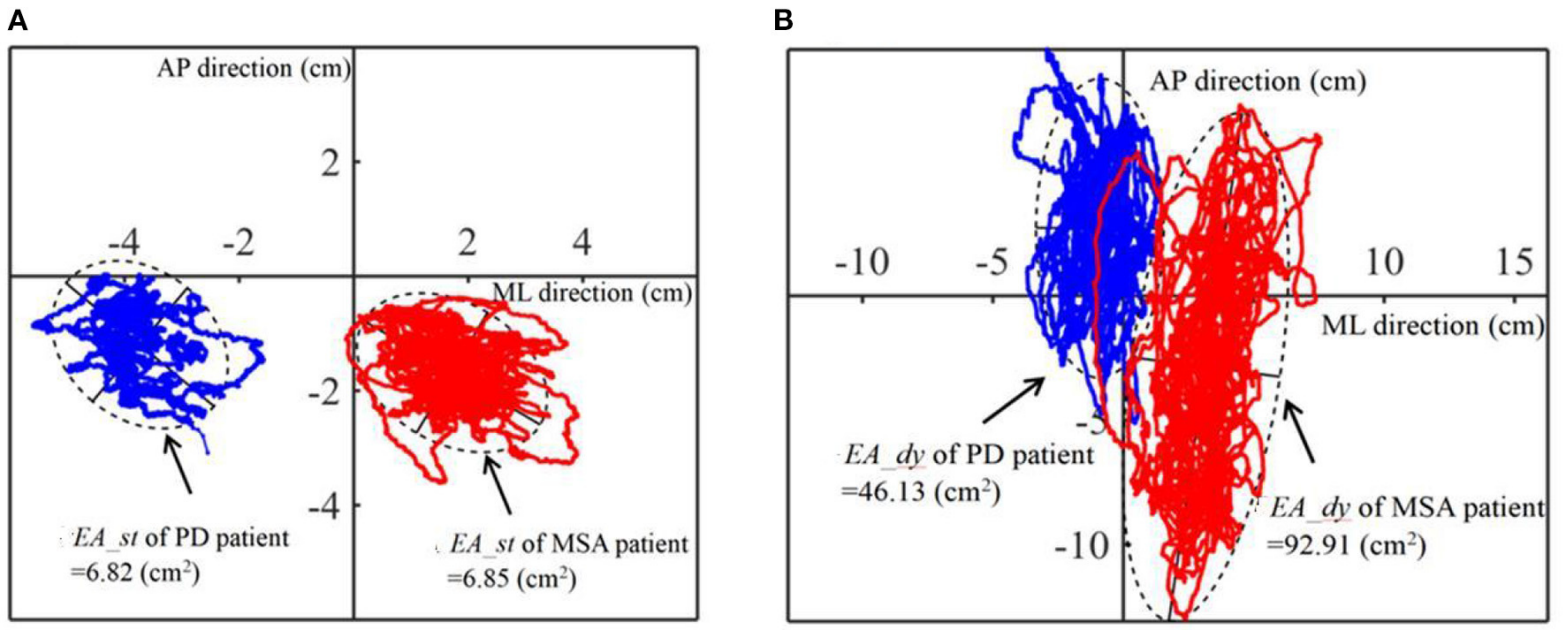
Schematic diagram of parameter *EA* of one PD patient and one MSA patient. **(A)** Static balance experiment. **(B)** Dynamic balance experiment. PD, Parkinson's disease; MSA, multiple system atrophy; COP, the center of pressure; ML, medial-lateral; AP, anterior-posterior.

Figure 2A shows the typical elliptical area (*EA_st*) of a sample PD patient and a sample MSA patient with EO in the static balance experiment. The value of the blue elliptical area (the patient with PD) is similar to the value of the red elliptical area (the patient with MSA). Figure 2B depicts the elliptical area (*EA_dy*) of the same patient with PD and the same patient with MSA in the dynamic balance experiment. The *EA_dy* value of the patient with PD (46.13 cm^2^) was significantly smaller than that of the patient with MSA (92.91 cm^2^).

**Figure 3:**
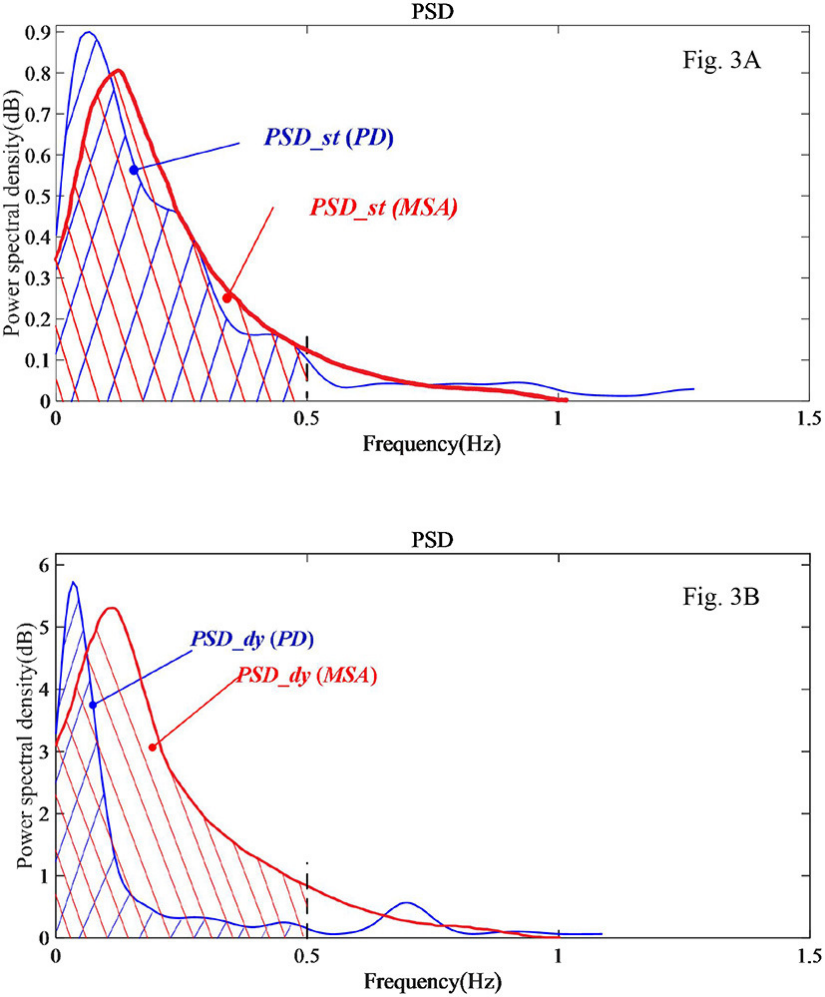
PSD analysis diagram of a sample PD and a sample MSA patients' COP signal in the EO state. **(A)** Static balance experiment. **(B)** Dynamic balance experiment.

Figure 3 shows processed sample data of a patient with PD and a patient with MSA in the form of power spectral density of COP changes with the frequency of COP. The variable PSD is displayed in the figure as the integral area of the corresponding curve up to the 0.5 Hz frequency band. In Figure 3A of the static balance experiment, there is little difference in the integral area under the PSD curve (*PSD_st*) between the PD sample and the MSA sample. However, in Figure 3B of the dynamic balance experiment, a salient difference can be seen in the variable *PSD_dy* between the PD sample and the MSA sample.

Table 5 lists Spearman's rank correlation (rho) between the subject's posturographic variable and H&Y scale score. It can be seen that the dynamic balance variable sway path (*SP*_*dy*) in the MSA group is most relevant (rho = −0.484).

**Table 5:** Spearman's rank correlation (rho) between subjects' posturographic variables and H&Y scale score.

**State of EO**	**PD (*N =* 42)**		**MSA (*N =* 32)**		**MSA-C (*N =* 16)**		**MSA-P (*N =* 16)**	
**Variable**	**Rho**	**P-value**	**Rho**	**P-value**	**Rho**	**P-value**	**Rho**	**P-value**
**Static**								
*SD*_*st*	−0.088	0.611	0.14	0.462	0.373	0.189	0.122	0.654
*EA_st* (cm^2^)	−0.077	0.646	−0.154	0.417	0.656	0.011^*^	−0.017	0.95
*SP*_*st* (cm)	−0.007	0.966	0.219	0.571	0.372	0.19	−0.063	0.816
*PSD*_*Power*_*st*	0.174	0.318	0.192	0.621	0.194	0.1	0.094	0.2
**Dynamic**								
*SD*_*dy*	0.112	0.48	0.405	0.026^*^	0.302	0.295	0.011	0.968
*EA_dy* (cm^2^)	0.171	0.278	0.327	0.077	0.089	0.763	−0.075	0.783
*SP*_*dy* (cm)	0.119	0.453	0.484	0.007^**^	0.195	0.504	0.093	0.731
*PSD _dy*	0.04	0.8	0.472	0.008^**^	0.337	0.239	0.137	0.613

^*^Indicates p-value < 0.05, ^**^Indicates p-value < 0.01. PD, Parkinson's disease; MSA, multiple system atrophy; EO, eyes-open, COP, the center of pressure; st, static; dy, dynamic.

## Discussion and conclusion

In this study, the differences in postural balance between Parkinson's disease and MSA were studied. The posturographic characterization of PD and MSA groups under both the static balance and the dynamic balance conditions at the state of EO was represented by three spatiotemporal parameters, namely, *SD, SP*, and *PEA*, and one frequency domain variable, namely, *PSD*. The four parameters in the static balance experiment show no significant differences between the PD and the MSA groups at the state of EO. However, significant differences in the four parameters between the PD and the MSA groups were presented in the dynamic balance experiments.

The posturographic variables, such as standard deviation (*SD*), sway path (*SP*), and elliptical area (*EA*), are all spatiotemporal measures of the COP trajectory. A previous study reported that in the EC state, the elliptical area of the COP displacement trajectory with patients with MSA under static standing was statistically larger than that with patients with PD. The different results under EO and EC conditions indicated that the effect of vision block on postural instability in patients with MSA is greater than that in patients with PD (18). The larger elliptical area covering the COP position trajectory usually indicates that the body has a poorer ability for postural control (36). The current results showed no significant differences between the MSA group and the PD group under the static balance condition, which implies that such spatiotemporal variables are normally inadequate to differentiate the postural control abilities between patients with MSA and those with PD during quiet standing in the EO state. In the dynamic balance experiment, the patients needed to respond to the coordination with the swinging platform along the ML direction. The experimental results show that the spatiotemporal variables of patients with MSA are statistically significantly larger than those of the PD group. Since patients with MSA usually have a broad-based stance and more instability in the ML direction, it is more difficult for patients with MSA to adjust and coordinate balance in the ML direction under interference, thus resulting in larger spatiotemporal variables. Other clinical studies also reported that the feature of ML balance impairment from various atypical parkinsonians like MSA can be revealed from simple observation tests (23). But in the early stages of the patient's illness, some subclinical posture instability could be difficult to evaluate without objective assessments (17). This study may provide an objective measure to assist these observation tests.

The power spectra of the COP time series provided more information about the structure of the COP signal. The power spectral density of the COP signals is mainly concentrated below the 0.5 Hz frequency range, which is represented by the variable *PSD*. In our study, the results show that the value of *PSD*_*dy* for the MSA group was statistically higher than that of the PD group in the dynamic balance experiment, whereas no statistical differences in *PSD_st* were seen between those two groups in the static balance experiment. It can be deduced that COP oscillations were more exacerbated in MSA than PD groups in the dynamic standing along the ML direction. This is possibly caused by a more widespread degeneration in MSA than in PD groups. The frequency below 0.5 Hz can reflect an oscillation that was part of the descending drive to the motor neuron pool (37, 38). A larger oscillation in the lower frequency band indicates increased activity within the relevant postural subsystem, either due to pathology or compensatory efforts. When the sway amplitude in the ML direction exceeds a threshold range, the intermittent control mechanism will be triggered (39). It has been reported that COP oscillations below 0.5 Hz were exacerbated in an early and moderate PD relative to the healthy group in the state of EO (40). Since all the participants were in an early or moderate stage of the disease, balance impairments in the ML direction were not obvious and could not be discriminated against during static standing. The coordinative disorder was amplified when standing on the dynamic platform, resulting in a significant difference in the variable between the two groups of patients.

Furthermore, the four parameters were compared between MSA-C and MSA-P patients. The four COP parameters of MSA-C patients were statistically larger than those of MSA-P patients in the dynamic balance experiment. Generally, the cerebellum is severely damaged in MSA-C patients, which can result in a worse postural control ability compared with MSA-P patients. The variable *PSD*_*dy* shows the largest difference between MSA-C and MSA-P, with a significant difference (*p*-value = 0.016). This is also consistent with the previous study by Li et al., who found that MSA-C can be effectively distinguished from MSA-P by relying on PSD (41). The staging of the functional disability associated with Parkinson's disease is commonly evaluated through H&Y scales (42). The H&Y scales have been validated not only in PD but also in MSA for the assessment of severity and disability. In our study, the participants in the experiment are in the early or middle stages, and the corresponding H&Y scale is 1–3 levels. Spearman's rank correlation was performed between the four posturographic variables and H&Y scales for both the PD and MSA groups. *SP*_*dy* (rho = 0.484, *p*-value = 0.007) was found to be the most relevant variable in the MSA group. This parameter may be used as a marker for studying the degree of disability in MSA.

We studied the quantitative posturographic parameters of body balance in a PD group and an MSA group under the conditions of static balance and dynamic balance. The postural balance indices with significant differences in the dynamic balance condition reflected that the postural control ability of patients with MSA is poorer in the ML direction compared to patients with PD. Those indices can be used to help distinguish between patients with MSA and patients with PD.

## Data availability statement

The data presented in the study are deposited in the Figshare website repository, accessible with the following link, https://doi.org/10.6084/m9.figshare.19633638.v6.

## Ethics statement

The studies involving human participants were reviewed and approved by the Ethics Committee of Shanghai Ruijin Hospital (Approval Number LWEC2019017). The patients/participants provided their written informed consent to participate in this study.

## Author contributions

KC and JL got the original ideas and designed the study. WB and PL performed the experiments. WB, PL, YY, and KC ran the statistics. WB and KC drafted the manuscript. KC and JL supervised the study. All authors contributed to the article and approved the submitted version.
