# Supplementary material for: Dynamic postural balance indices can help discriminate between patients with multiple system atrophy and Parkinson's disease
Source: Front Neurol. 2023 Jan 9;13:1089439. doi: 10.3389/fneur.2022.1089439 (PMC9868697; doi:10.3389/fneur.2022.1089439)
Supplement: Supplementary file 1 [file Data_Sheet_1.docx]

Supplementary Materials

We analysed COP position in the anterior-posterior direction (AP, noted as *y* axis), and medial-lateral direction (ML, noted as *x* axis). The velocity series were obtained by differentiating the position series.

The coordinate equation was as follows:

$x=x'-\overline{x}$ (1)

$y=y'-\overline{y}$ (2)

where *x* and *y* represent COP positions in ML direction and AP direction after the offset was removed respectively, *x*' and *y*' were the original COP displacements in ML and AP directions,$\overline{x}$and$\overline{y}$are the offsets in corresponding directions.

1. The standard deviations of COP position in ML directions, expressed as *SD* was calculated as follows:

$SD=\sqrt{\frac{1}{N}\sum_{i=1}^{N} \left(x_{i}-\overline{x}\right)}$ (3)

1. The prediction ellipse area (*EA*) was defined as the area of ellipse that encloses 95% the points of COP movement positions, and was calculated by the following steps:

$C=\left(\begin{matrix} \sigma_{11} & \sigma_{12} \\ \sigma_{21} & \sigma_{22} \end{matrix}\right)$ (4)

$\lambda_{1}=\frac{\sigma_{11}+\sigma_{22}+\sqrt{\left(\sigma_{11}-\sigma_{22}\right)^{2}+4\sigma_{12}^{2}}}{2}$ (5)

$\lambda_{2}=\frac{\sigma_{11}+\sigma_{22}-\sqrt{\left(\sigma_{11}-\sigma_{22}\right)^{2}+4\sigma_{12}^{2}}}{2}$ (6)

$\kappa=\sqrt{-2\ln\left(1-\alpha\right)}=\sqrt{-2\ln0.05}$ (7)

$EA=\pi\times\kappa\sqrt{\lambda_{1}}\times\kappa\sqrt{\lambda_{2}}$ (8)

where$\sigma_{11}$and$\sigma_{22}$represented the variance of COP position in ML and AP direction respectively. $\sigma_{12}$and $\sigma_{21}$represented the covariance of COP position in ML and AP direction respectively. *C* was the eigenvalue of the covariance matrix. $\lambda_{1}$ denoted the first main component, and$\lambda_{2}$the second main component.$\kappa$was the confidence interval.$\kappa\sqrt{\lambda_{1}}$and $\kappa\sqrt{\lambda_{2}}$represented major and minor axes values of the confidence ellipse.

1. The sway paths of the COP positions (*SP*) in ML direction were obtained by：

$SP=\sum_{\text{i=1}}^{N} \sqrt{\left(x_{i+1}-x_{i}\right)^{2}}$ (9)

1. Variable of power spectra density (PSD) was calculated by the following equations.

$PSD=\sum_{m=i}^{j} m\Delta fG\left[m\right]$ (10)

where power was the integrated area of PSD from particular spectra. $G\left[m\right]$was the discretized power spectrum density function ,$\Delta f$meant the frequency increment, $i$ and $j$ represented the lowest and highest discrete frequency values for integration, which was from 0.01Hz and 0.5Hz .
